# Single-Cell Cloning of Breast Cancer Cells Secreting Specific Subsets of Extracellular Vesicles

**DOI:** 10.3390/cancers13174397

**Published:** 2021-08-31

**Authors:** Mohsen Fathi, Robiya Joseph, Jay R T. Adolacion, Melisa Martinez-Paniagua, Xingyue An, Konrad Gabrusiewicz, Sendurai A. Mani, Navin Varadarajan

**Affiliations:** 1 Chemical and Biomolecular Engineering Department, University of Houston, 4726 Calhoun Rd, Houston, TX 77204, USA; mfathi@uh.edu (M.F.); jtadolacion@up.edu.ph (J.T.A.); mamart51@Central.uh.edu (M.M.-P.); xingyuean@gmail.com (X.A.); 2 Department of Translational Molecular Pathology, University of Texas M.D. Anderson Cancer Center, 2130 W Holcombe Blvd, Houston, TX 77030, USA; RJoseph7@mdanderson.org (R.J.); mani@mdanderson.org (S.A.M.); 3 Department of Chemical Engineering, College of Engineering, University of the Philippines Diliman, Quezon City 1101, Philippines; 4 Department of Neurosurgery, University of Texas M.D. Anderson Cancer Center, 1400 Holcombe Blvd, Houston, TX 77030, USA; konrad.gabrusiewicz@gmail.com

**Keywords:** single-cell analysis, metastasis, exosomes, macrophage

## Abstract

**Simple Summary:**

Extracellular vesicles (EVs) are a pivotal mechanism for long-distance intercellular communication and facilitate the stable transport of biological information. Conventional methods for profiling EVs are focused on the biological cargo obtained from large populations of cells and cannot map the secretion of specific subsets of EVs onto their cell of origin. We developed a high-throughput single-cell cloning method that can identify the kinetics of secretion of specific subsets of EVs. With the aid of this methodology, we illustrate that secretion of specific subsets of EVs can be an inheritable property of cancer cells. Our single-cell methodology enables the direct integration of EV secretion with multiple cellular functions and can enable new insights into cell and disease biology.

**Abstract:**

Extracellular vesicles (EVs) mediate communication in health and disease. Conventional assays are limited in profiling EVs secreted from large populations of cells and cannot map EV secretion onto individual cells and their functional profiles. We developed a high-throughput single-cell technique that enabled the mapping of dynamics of EV secretion. By utilizing breast cancer cell lines, we established that EV secretion is heterogeneous at the single-cell level and that non-metastatic cancer cells can secrete specific subsets of EVs. Single-cell RNA sequencing confirmed that pathways related to EV secretion were enriched in the non-metastatic cells compared with metastatic cells. We established isogenic clonal cell lines from non-metastatic cells with differing propensities for CD81^+^CD63^+^EV secretion and showed for the first time that specificity in EV secretion is an inheritable property preserved during cell division. Combined in vitro and animal studies with these cell lines suggested that CD81^+^CD63^+^EV secretion can impede tumor formation. In human non-metastatic breast tumors, tumors enriched in signatures of CD81^+^CD63^+^EV have a better prognosis, higher immune cytolytic activity, and enrichment of pro-inflammatory macrophages compared with tumors with low CD81^+^CD63^+^EVs signatures. Our single-cell methodology enables the direct integration of EV secretion with multiple cellular functions and enables new insights into cell/disease biology.

## 1. Introduction

Extracellular vesicles (EV) comprise a fundamental mechanism of intercellular communication across distant cells and serve to transport biological molecules such as lipid, nucleic acids, and proteins. Encapsulation of molecules into EVs fundamentally alters their stability, transport, and trafficking, and characterizing the secretion of EVs cargo from cells is of great interest in fundamental cell biology and for targeted drug delivery [1,2,3,4].

In the context of cancer, EVs are known to affect a variety of biological events that promote tumor progression such as angiogenesis [5,6], invasion [7], evasion of immune surveillance [8,9], and drug resistance [10]. EVs from highly metastatic melanoma tumors promoted vascular permeability and contributed to the formation of the pre-metastatic niche [11]. Additionally, EVs can transfer antigens and enhance the immune response by activating T cells and NK cells [12,13]. Due to their stability, they have great potential in cancer diagnosis [14,15] and treatment [16]. Mapping the dynamic secretion of EVs at the cellular level and the heterogeneity of the EVs secreted by different cells within the same population can advance our understanding of cancer.

From an analytical standpoint, the size of EVs (40–150 nm) is in between the size of proteins and cells. Several analytical methods including nanoparticle tracking analysis (NTA) [17], electron microscopy [18], flow cytometry [18,19], microfluidic devices [20], and Western blotting [21], have been widely used for the characterization of EVs, some even with the sensitivity of detecting individual EVs. Unfortunately, however, these EVs are derived from culturing billions of cancer cells and thus represent an averaging of EVs secreted by all cells. From the standpoint of disease biology, this is suboptimal since these might reflect supraphysiological concentrations of these EVs. Second, since tumors are heterogeneous populations, these approaches mask the inherent differences in EV secretion between individual cells, and mapping the direct relationship between EV secretion and tumorigenic potential is not feasible. Third, while these methods are excellent for profiling EVs in a marker-free manner (high yield) they suffer from low specificity in being able to map specific subsets of EVs onto subsets of cancer cells. Not surprisingly, recent advances in microfabrication have revealed that the rate of EV secretion from single cells can be heterogenous [22,23,24,25]. Despite this progress, however, technological hurdles have prevented us from answering many questions at the single-cell level including (1) heterogeneity of the short-term dynamics of secretion of EVs or subsets of EVs, (2) whether EV secretion is an inheritable property preserved upon cell division, and (3) whether there is a difference in tumorigenic potential between isogenic tumor cells with differences in the rate of EV secretion.

We report a high-throughput single-cell technique for the dynamic quantification of EV secretion from single cells. We utilized the 4T1 and 67NR syngeneic mouse mammary tumor models since these are well-validated, clinically relevant models with vastly different potential for metastasis. 4T1 spontaneously metastasizes to multiple sites, whereas 67NR is incapable of metastasis and is restricted to the formation of the primary tumor [26]. By tracking the dynamics of EV secretion, we demonstrate that in both cell lines, the dominant secretor cells are capable of continuous secretion over short time intervals (6–24 h). Surprisingly, the non-metastatic 67NR cells secreted more CD81^+^CD63^+^EVs per cell than 4T1 cells, and this result was consistent with scRNA-seq of the same cells, showing an enrichment of the ALIX-Syndecan-Syntenin pathway. Although the secretion of CD81^+^CD63^+^EVs from 67NR clones caused an increase in proliferation and migration in vitro, the tumor growth was inhibited in vivo. Analysis of The Cancer Genome Atlas (TCGA) data unexpectedly illustrated that the secretion of CD81^+^CD63^+^EVs is associated with better overall survival of non-metastatic patients, which was induced by higher secretion of IFN-γ, higher infiltration of Th1 cells, the polarization of M1 macrophages, and suppression of the IL6ST/STAT3 pathway. More broadly, the CD81^+^CD63^+^EV secretion signatures are also associated with a better prognosis in non-metastatic melanoma but a worse prognosis in non-metastatic lung cancers.

## 2. Materials and Methods

### 2.1. Cell Culture

4T1 and 67NR cells were purchased from ATCC. We cultured cells in RPMI 1640 supplemented with 10% Fetal Bovine Serum (FBS), 1% L-glutamine, HEPES, and penicillin-streptomycin. We tested all cells for mycoplasma contamination using real-time PCR. For experiments that we used EV-free complete media, we supplemented the RPMI 1640 with EV-free FBS instead of FBS.

### 2.2. Functionalization of Beads with Capture Antibody

We washed 10^5^ LumAvidin beads (Luminex, catalog number L100-L115–01) in Phosphate-Buffered Saline (PBS) with 1% Bovine Serum Albumin (BSA) and incubated the beads with 3.5 µg/mL biotinylated anti-CD81 antibody (BioLegend, catalog number 104903-clone Eat-2), biotinylated anti-CD63 antibody (BioLegend, catalog number 143918-clone NVG-2), and/or biotinylated anti-CD9 antibody (BioLegend, catalog number 124803-clone MZ3) at the room temperature for 40 min. Then, after washing beads thrice in PBS with 1% BSA, we resuspended them in 120 µL of PBS with 1% BSA.

### 2.3. Single-Cell EV Detection Assay

To perform the single-cell assay for the detection of EVs, we prepared the nanowell array and functionalized beads, as described above. We labeled 67NR or 4T1 cells with PKH67 dye (Sigma-Aldrich, St. Louis, MO, USA, catalog number PKH67GL-1KT) as directed by the manufacturer. We loaded labeled cells and functionalized beads sequentially on the nanowell array. We covered the nanowell with EV-free complete media and imaged it at the initial time point, incubating at 37 °C. Every two hours, we incubated the nanowell array with 4 µg/mL PE anti-CD63 antibody (BioLegend, Santiago, CA, USA, catalog number 143903-clone NVG-2), PE anti-CD81 antibody (BioLegend, catalog number 104905-clone Eat-2), and/or PE anti-CD9 antibody (BioLegend, catalog number 124805-clone MZ3) for 45 min at 37 °C. We subsequently washed the nanowell array three times in PBS with 1% BSA and performed imaging using microscopy. After each imaging, we returned the nanowell to the incubator at 37 °C. We acquired all images by Zeiss Axio Observer Z1 microscope equipped with 20×/0.8 NA objectives and a Hamamatsu Orca Flash v2 camera.

### 2.4. Secretion Analysis of Single-Cell EV Detection Assay

We analyzed the TIFF images from microscopy using ImageJ, as outlined in Figure S3. Briefly, for all images at each time point, we segmented the images into cells and beads and determined the ratio of the number of cells to the number of beads in each well. After identification of wells with a single bead and a single cell, we tracked the wells across time points. We background-corrected the CD63 pixel values and compared the pixel values between the bead and non-overlapped pixels on the cells using a two-tailed *t*-test. Based on the average intensity and the *p*-value calculated, we classified the single cells as either secretor (high secretor) or non-secretor (low secretor) cells.

### 2.5. Kinetic Analysis of Single-Cell EV Detection Assay

Using the wells containing a single bead and a single cell identified in the secretion analysis of single-cell EV detection assay, we selected the wells which were detected in all the time points for the kinetic analysis. To determine the behavior of the cells between time points, we performed a two-tailed *t*-test on the CD63 pixel values of the bead between two consecutive time points. We chose an increase in intensity with a *p*-value below 0.01 as the criterion for a significant change in the secretion behavior of the cell.

### 2.6. Establishment of Clonal Cell Lines

We retrieved the secretor and non-secretor single cells using a micromanipulator (ALS, CellCelector) equipped with 50 μm glass capillaries. We transferred single cells to a 96-well plate containing complete media. We monitored the single cells and cultured them in complete media until they proliferated to 24 population doublings.

## 3. Results

### 3.1. Establishing a Single-Cell Method for Mapping Cell Function Onto EVs Secretion

We sought to establish a method based on open nanowell arrays for identifying the secretion of EVs at the single-cell level based on functionalized beads. The open nanowell arrays, as we have previously demonstrated, enable the longitudinal tracking of the dynamic properties of the cells without the confounding artifacts of encapsulation [27].

For the identification of EVs, the expression of transmembrane proteins, CD63, CD81, and CD9 on the surface of EVs, has been widely used [28]. However, the expression of these surface proteins is not uniform across all EVs which ends up with a heterogeneous pool of EVs. Since the current ultracentrifugation methods lack specificity for the isolation of EVs, we sought to take advantage of antibodies specific for EV surface markers to capture these EVs secreted from single cells onto functionalized beads. To confirm the capability of functionalized beads for the isolation of EVs, we compared the size of EVs isolated by ultracentrifugation with EVs isolated using functionalized beads. Nanoparticle tracking analysis (NTA) confirmed that the EVs isolated using ultracentrifugation had a median diameter of (132 ± 6) nm (Figure S1A). To test the functionalized beads, we used a transwell assay [29,30,31] in which we incubated the cells in the upper chamber and functionalized beads at the bottom (Figure S1B). After 48 h, the EVs isolated using this procedure displayed the expected morphology and size as observed by transmission electron microscopy (TEM) (Figure S1C). Collectively, these results suggest that the bead-based immunosandwich utilizing functionalized beads can be used to capture EVs from cells and could be used for single-cell assays.

**Figure 1:**
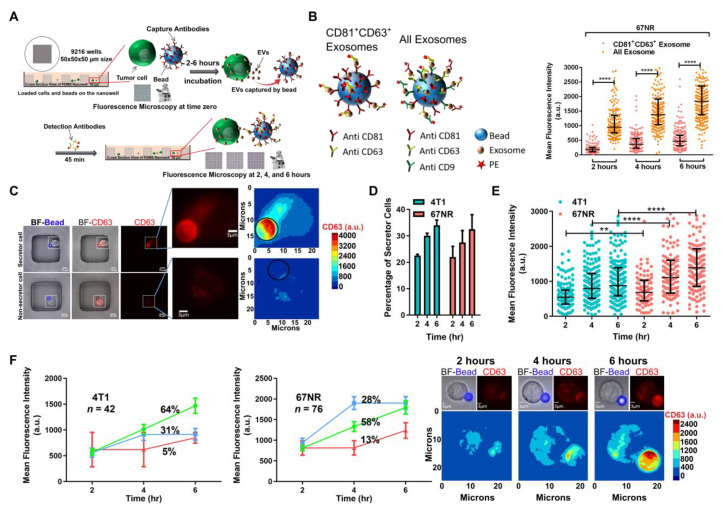
High-throughput single-cell assay for monitoring EV secretion. (**A**) The overall workflow of the single-cell assay. Cells and anti-CD81 conjugated beads are loaded on the nanowell and incubated for 2–6 h. The entire array is incubated with fluorescently labeled antibodies against CD63 and imaged using microscopy. (**B**) Schematic of immunoassays for profiling pan EVs using a cocktail of CD63, CD81, and CD9 antibodies and subset of CD81^+^CD63^+^EVs using CD81 for capture and CD63 for detection. Each dot represents a single cell with its mean fluorescence which is the intensity of PE anti-CD63 antibody for CD81^+^CD63^+^EVs or intensity of PE anti-CD63, PE anti-CD81, and PE anti-CD9 antibodies for pan EVs. **** *p* < 0.00001. (**C**) Representative images of individual nanowells containing 67NR cells with different EV secretion capacities. The insets show single cells and the contour map of CD63 (EVs) intensity. (**D**) Comparison of the frequency of CD81^+^CD63^+^EVs secreting cells between 67NR (non-metastatic) and 4T1 (metastatic) breast cancer cells (mean ± SEM). (**E**) The rate of secretion of CD81^+^CD63^+^EVs by 67NR cells is higher than that of 4T1 cells at two, four, and six hours. Each dot represents a single cell with the median and quartiles of CD63 (EVs) intensity shown across all cells. A representative example from three independent repeats is shown. ** *p* < 0.001 and **** *p* < 0.00001. (**F**) The kinetics of EV secretion from single cells. The three subpopulations corresponding to (1) continuous secretion (green), (2) secretion initiated at two hours (blue), and (3) delayed secretion at four hours (red) are shown as trend lines (mean ± SEM). Representative images and contour maps of a single cell showing a continuous increase of CD63 intensity on the surface of the bead.

To analyze the secretion of EVs from single cells, we utilized a custom nanowell array containing 9216 wells, and co-incubated functionalized beads and cancer cells (Figure 1A and Figure S2). To profile a broad collection of EVs, we used a cocktail of antibodies targeting CD63, CD81, and CD9 (pan EVs) for both capturing and detecting the EVs secreted from 67NR cells. At two-hour intervals, we added the fluorescently tagged antibodies and imaged the entire nanowell array. We observed a steady increase in the number of cells secreting EVs from 2–6 h (Figure 1B). We next sought to identify the specificity/heterogeneity of EV secretion and profiled 67NR cells secreting CD81^+^CD63^+^EVs. We chose these two markers since they have a well-documented but controversial role in breast cancer metastasis and epithelial to mesenchymal (EMT) transition [32,33]. Comparison of CD81^+^CD63^+^EVs versus pan EVs showed that 67NR cells secrete 4-fold less CD81^+^CD63^+^EVs compared with the pan EVs secreted by the same cells (Figure 1B). These results established that depending on the markers used for capture and staining, our single-cell methodology can identify cells secreting broad classes of EVs or a specific subset of EVs.

To map the functional heterogeneity of cells to their ability to secrete specific subsets of EVs, we focused on cells secreting CD81^+^CD63^+^EVs (Figure 1C). We compared the frequency of single cells secreting EVs between 67NR, and the isogeneic, metastatic breast cancer cell line, 4T1. At each of the time points tested—two, four, and six hours—there was no difference in the frequency of single cells secreting EVs, comparing 4T1 and 67NR (Figure 1D). Within all cells that secreted EVs, we also compared the number of EVs secreted per cell across 4T1 and 67NR single cells. Surprisingly, single cells from the non-metastatic cell line 67NR secreted more CD81^+^CD63^+^EVs per cell at each of the time points profiled (Figure 1E). Tracking the kinetics of EV secretion in individual 4T1 and 67NR cells during a six-hour period revealed three major classifications for the cells: (1) a major subpopulation of cells that showed continuous secretion, (2) a subpopulation of cells that showed secretion at two hours, then subsequently stopped secreting, and (3) cells with delayed secretion starting at four hours (Figure 1F). Taken together, these results established that while the overall frequencies of cells secreting EVs are not necessarily different between metastatic and non-metastatic cell lines, individual cells exhibited asynchronous release of EVs. These results also indicate that non-metastatic 67NR cells can secrete more CD81^+^CD63^+^EVs per cell in comparison with metastatic 4T1 cells, a characteristic that is masked by routine ultracentrifugation procedures.

### 3.2. Single-Cell RNA-Sequencing Illustrates That 67NR Cells Are Enriched in EV Secretion Pathways Compared with 4T1 Cells

**Figure 2:**
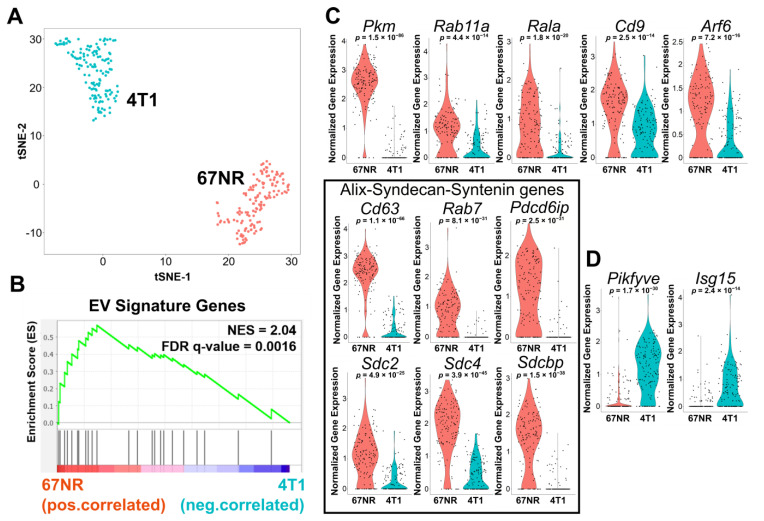
Comparison of 4T1 and 67NR cells by scRNA-seq. (**A**) t-SNE plot of 67NR (non-metastatic) and 4T1 (metastatic) breast cancer cells clusters analyzed by scRNA-seq. (**B**) Gene set enrichment analysis (GSEA) of the core EV gene signature studies within 67NR cells compared with 4T1 cells. The core gene signature was based on a previously described set of genes known to be involved in EV secretion (Table S1). (**C**) Violin plots of the EV secretion genes upregulated in 67NR in comparison with 4T1. Each dot represents one single cell. The black box highlights genes associated with the ALIX-Syndecan-Syntenin pathway. (**D**) Violin plots of EVs inhibitors, *Pikfye*, and *Isg15*, upregulated in 4T1 cells in comparison with 67NR cells. Each dot represents one single cell.

To gain further mechanistic insights into the pathways that can support the increased EV secretion capacity of 67NR cells in comparison with 4T1 cells, we performed single-cell RNA-sequencing (scRNA-seq). After data processing (see Methods), the final scRNA-seq dataset used for analysis had an average of 3386 unique genes per cell and 35,604 transcripts (Figure S4A). Dimensionality reduction using t-distributed stochastic neighbor embedding (t-SNE) showed a clear separation between the cells comprising each cell line (Figure 2A). Hierarchical clustering indicates that a set of 1647 differentially expressed genes (≥2-fold change) distinguishes the two cell types (Figure S4B). ScRNA-seq confirmed that several markers associated with epithelial-mesenchymal transition (EMT) including vimentin (Vim), fibronectin (Fn1), and Axl receptor tyrosine kinase (Axl) were increased in 67NR cells (Figure S4C). In contrast, several matrix metalloproteinases associated with invasion, including Mmp9 and Mmp14, were increased in 4T1 cells (Figure S4D).

To test the correlation between the functional single-cell EV secretion assay and the transcriptional signatures, we established a core gene signature using a previously described set of genes known to be involved in EV secretion (Table S1) [34]. Gene set enrichment analysis (GSEA) comparing 4T1 and 67NR confirmed that 67NR cells were positively correlated with EV secretion signatures (Figure 2B,C). The core set of genes in the GSEA that showed high discrimination between 4T1 and 67NR cells mapped to the known ALIX-Syndecan-Syntenin pathway [35]. The pathway genes consisting of tetraspanins (*Cd63*), Rab7, apoptosis-linked gene 2-interacting protein X (*Pdcd6ip*), syndecans (*Sdc2*, *Sdc4*), and syntenin (*Sdcbp*) were enriched in 67NR cells compared with 4T1 cells (Figure 2C). By contrast, two proteins that are known EV secretion inhibitors, Pikfyve and Isg15, were significantly expressed in 4T1 cells but not in 67NR cells (Figure 2D) [36,37]. We performed independent verification of these results by reanalyzing population-level RNA-seq data on these same cell lines (GSE104765) [38]. These data also confirmed the higher expression of *Cd63*, *Rab7*, *Sdc2*, *Sdc3*, and *Sdcbp* in 67NR cells in comparison with 4T1 cells (Figure S4E). Collectively, these results from transcriptional profiling further advanced our observation that non-metastatic breast cancer cells can secrete more EVs than metastatic breast cancer cells, and suggest that the ALIX-Syndecan-Syntenin pathway supports this function.

### 3.3. EV Secretion Is an Inheritable Property during Short-Term Culture of Cancer Cells

**Figure 3:**
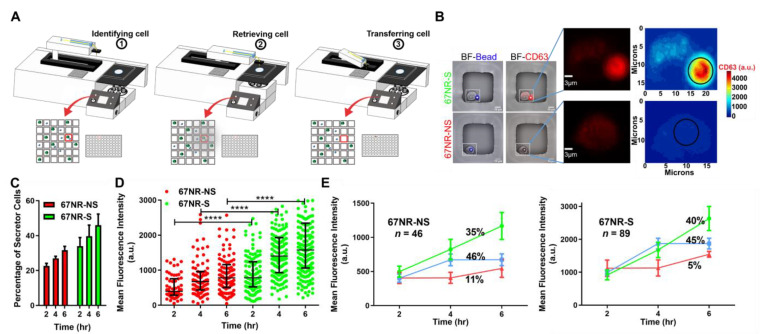
Establishment and validation of 67NR secretor (S) and non-secretor (NS) clonal cell lines. (**A**) Schematic of the overall workflow for the imaging and retrieval single cells secreting EVs with the aid of an automated micromanipulator. (**B**) Representative images of 67NR secretor and non-secretor single cells before retrieval. The insets show single cells and the contour map of CD63 (EVs) intensity. (**C**) Comparison of the frequency of cells secreting EVs within expanded populations of 67NR-NS and 67NR-S cells (mean ± SEM). (**D**) The rate of secretion of CD81^+^CD63^+^EVs by cells within the 67NR-S population is higher than the rate of secretion by cells within the 67NR-NS population at two, four, and six hours. Each dot represents a single cell with the median and quartiles of CD63 (EVs) intensity shown in all cells. A single representative experiment from four independent repeats is shown. **** *p* < 0.00001. (**E**) The kinetics of EV secretion from individual cells that comprise the 67NR-NS and 67NR-S populations. The three subpopulations corresponding to (1) continuous secretion (green), (2) secretion initiated at two hours (blue), and (3) delayed secretion at four hours (red) are shown as trend lines (mean ± SEM).

Our combined functional and transcriptional data illustrated that 67NR cells are proficient in CD81^+^CD63^+^EV secretion. We next wanted to investigate the impact of CD81^+^CD63^+^EV secretion on the functional properties of the 67NR tumor cells. We established a simple bioanalytical process to image cells secreting CD81^+^CD63^+^EVs using nanowell arrays and microscopy, performed automated segmentation and identification of secretor and non-secretor cells, and used an automated micromanipulator to retrieve single cells to establish clonal cell lines (Figure 3A, Video S1). Since we wanted to ensure that long-term culture did not alter the properties of the cells, we grew the cells to no more than 24 population doublings. For the majority of the cells picked (20 out of 27), we were able to establish clonal cell lines, classified as secretor (cell population labeled as S, secretor, if the cell of origin was a secretor) and non-secretor (cell population labeled as NS, non-secretor, if the cell of origin was a non-secretor) [Figure 3B].

We tested the ability of single cells derived from these expanded populations to secrete CD81^+^CD63^+^EVs using our single-cell assay. Consistently, across all six cell lines tested (three secretor lines and three non-secretor lines), the frequency of single cells secreting CD81^+^CD63^+^EVs was higher among the 67NR-S cell lines in comparison with the 67NR-NS cell lines (Figure 3C). Within all cells that secreted EVs, comparisons of the number of EVs secreted per single cell as a function of time (two, four, and six hours) confirmed that the 67NR-S cell lines were composed of individual cells with high rates of EV secretion (Figure 3D). The numbers of EVs secreted by single cells from the 67NR-NS cell lines and the 67NR-S cell lines were, respectively, lower and higher than the parental unsorted 67NR cell line (Figure S5A). Kinetic analysis of the dynamics of EV secretion in these cell lines revealed two dominant subpopulations: (a) continuous secretors and (b) cells with a delayed secretion that stopped secretion after 4 h (Figure 3E). The asynchronous release of EVs from cells from these subpopulations mirrors the parental population (Figure 1F) and could arise from differences in the activity of kinases such as Ras or the differential activity of the tumor suppressor p53 that alters the cell cycle and directly influences proteins involved in EV secretion such as CHMP4C [39,40,41]. We confirmed the specificity of our cell lines with regard to EV secretion by comparing the secretion of CD81^+^CD63^+^EVs and the pan EVs. As expected, despite the higher secretion of CD81^+^CD63^+^EVs from the 67NR-S cell line, there was no significant difference in secretion of pan EVs between 67NR-S and 67NR-NS cell lines (Figure S5B). Taken together, these results establish that the secretion of EVs is inheritable during cell division, and the specificity of EVs is also maintained during cell division. This observation allowed us to investigate the functional consequences of cell lines secreting CD81^+^CD63^+^EVs.

### 3.4. Secretion of EVs Prevents the Tumor Formation in Non-Metastatic Cell Lines

**Figure 4:**
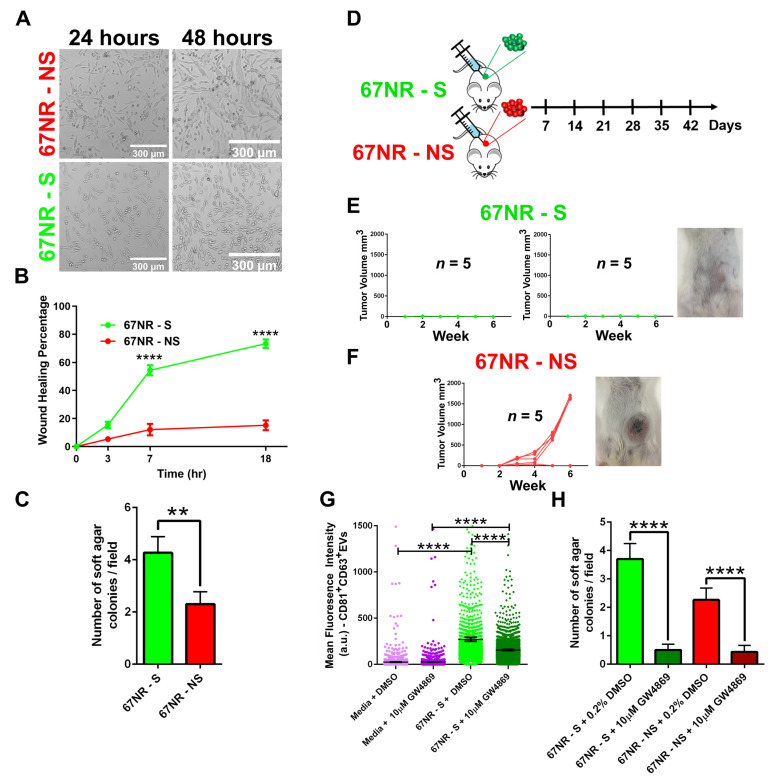
Assessing the functionality of 67NR-NS and 67NR-S cells. (**A**) The morphology of 67NR-NS and 67NR-S cell populations recorded using phase-contrast microscopy. (**B**) Wound healing assays illustrating the migration of 67NR-S and 67-NR-NS clonal cell populations (mean ± SEM, n = 6 for each cell line). Two-way ANOVA was used for comparison; **** *p* < 0.0001. (**C**) 67NR-S cell populations have a higher capacity to form colonies in comparison with 67NR-NS cell populations (mean ± SEM). The Mann–Whitney t-test was used for comparison; ** *p* < 0.01. (**D**) The design of mice experiments for comparing the efficacy of tumor formation by 67NR-S and 67NR-NS cell lines. (**E**) Tumor growth monitoring of BALB/c mice injected with 67NR-S clones (two clonal cell populations, five mice each). A representative image of a single mouse is shown. (**F**) Tumor growth monitoring of BALB/c mice injected with 67NR-NS clone (single clonal cell population, five mice). A representative image of a single mouse is shown. (**G**) Inhibition of EV secretion within 67NR-S clonal cell populations by 10 µM GW4869 compared with DMSO control using a 48 h transwell assay. Media containing either 10 µM GW4869 or DMSO were used as a negative control. Each dot represents CD63 (EVs) intensity on a single bead (mean ± SEM). The Mann–Whitney t-test was used for comparison; **** *p* < 0.0001. (**H**) Inhibition of colony formation in 67NR-S and 67NR-NS clonal cell populations upon treatment with 10 µM GW4869 (mean ± SEM). The Mann–Whitney t-test was used for comparison; **** *p* < 0.0001.

Since the expanded cell populations preserved the EV secretion property of the cell of origin, we investigated in vitro functions of the 67NR-S and 67NR-NS cell lines. Phase-contrast microscopy revealed differences in the morphology with 67NR-S cells being more elongated than 67NR-NS cells (Figure 4A). Migration is a key characteristic of cancer cells essential for metastasis. To test the migratory behavior of the 67NR-S and 67NR-NS cell lines, we performed a scratch wound assay [42]. 67NR-S cells were significantly more migratory than 67NR-NS cells (Figure 4B). To test the tumorigenicity potential of these cell lines, we used a colony formation assay to assess the anchorage independent growth of clonal cell lines as one of the parameters associated with tumorigenicity. 67NR-S cells formed 2-fold more colonies than the 67NR-NS cells in soft agar suspension cultures (Figure 4C). These in vitro data illustrate that the 67NR-S cells were more migratory and had enhanced tumorigenicity potential compared with 67NR-NS cells.

We have utilized syngeneic models to be able to understand the impact of EVs on both intrinsic growth potentials of the tumor and the impact of the host immune system. Parental 67NR cells are non-metastatic cells with a heterogeneous population and form primary tumors upon injection into mice. To determine the in vivo relevance of the secretion of CD81^+^CD63^+^EVs, we injected two 67NR-S and one 67NR-NS cell line into the mammary fat pad of BALB/c mice and monitored the tumor growth for six weeks (Figure 4D). None of the mice that received the 67NR-S cells developed tumors (Figure 4E). By comparison, however, 80% of the mice that received 67NR-NS cells formed large tumors by week six (Figure 4F). Taken together, these results illustrate that despite having high tumorigenicity and migratory potential in vitro, the 67NR-S cells failed to form tumors in vivo.

To directly link EV secretion to the rejection of tumors in vivo, we investigated the use of GW4869, a chemical inhibitor of EV biogenesis (pan EVs). Treatment of 67NR-S cells with GW4869 significantly inhibited secretion of pan EVs including CD81^+^CD63^+^EVs when profiled using the transwell EV capture assay (Figure 4G). Unfortunately, however, the treatment of 67NR-S cells with GW4869 almost completely abolished colony formation in a soft agar assay (Figure 4H), precluding its use in vivo. Collectively, studies with these non-metastatic breast cancer cells demonstrated that despite enhanced tumor-forming potential in vitro, cell lines secreting CD81^+^CD63^+^EVs were rejected in vivo, presumably due to the host immune system.

### 3.5. Secretion of EVs Improves the Survival in Non-Metastatic Breast Cancer Patients

**Figure 5:**
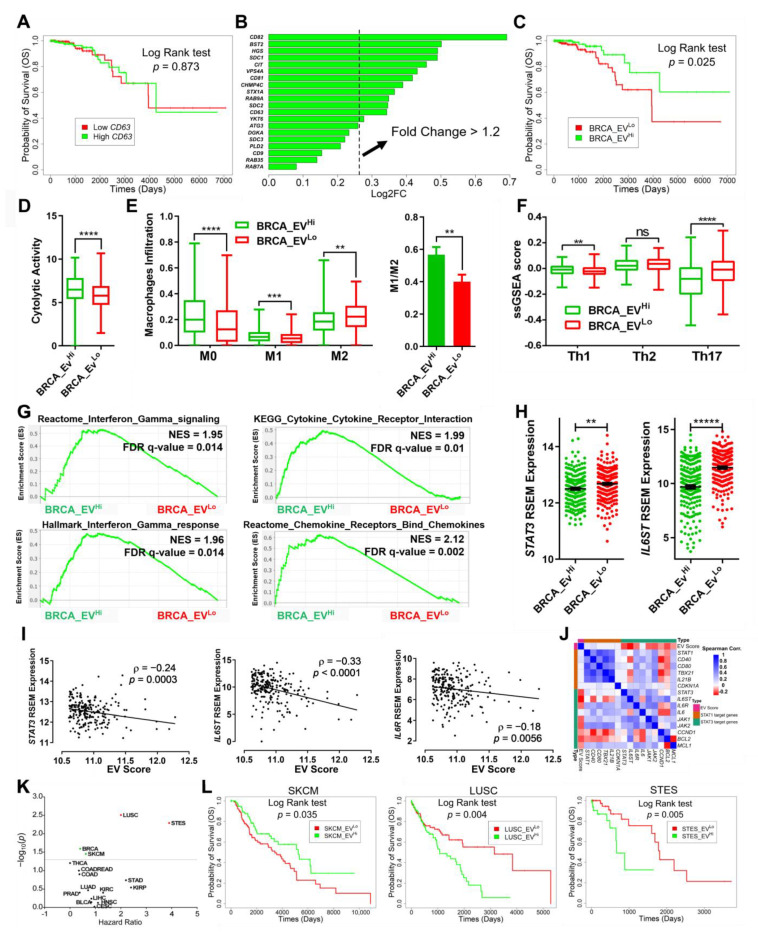
Increased EV secretion is correlated with better survival in non-metastatic breast cancer patients. (**A**) The overall survival of non-metastatic breast cancer patients (N0 and M0 in TNM staging system) divided by the median of CD63 expression. (**B**) Enrichment of the genes associated with CD81^+^CD63^+^EVs secretion in the BRCA_EV^Hi^ patients. The 13 genes with fold change > 1.2 were selected as the CD81^+^CD63^+^EV gene signature. (**C**) Differences in the survival of non-metastatic breast cancer patients stratified by the median expression of CD81^+^CD63^+^EV gene signature. (**D**) Cytolytic activity score of non-metastatic breast tumors comparing the BRCA_EV^Hi^ and BRCA_EV^Lo^ patients (mean ± SEM). **** *p* < 0.01. (**E**) Macrophage infiltration scores and ratio of M1/M2 macrophages for the BRCA_EV^Hi^ and BRCA_EV^Lo^ tumors determined by CIBERSORTx (mean ± SEM). ** *p* < 0.01, *** *p* < 0.001, **** *p* < 0.0001. (**F**) The immune score of T helper cells for BRCA_EV^Hi^ and BRCA_EV^Lo^ tumors was identified using the single sample Gene Set Enrichment Analysis (ssGSEA) (mean ± SEM). ns: nonsignificant, ** *p* < 0.01, **** *p* < 0.0001. (**G**) GSEA of interferon-gamma, cytokines/chemokines receptor interaction pathways comparing BRCA_EV^Hi^ and BRCA_EV^Lo^ tumors. (**H**) Normalized expression of STAT3 and IL6ST in BRCA_EV^Hi^ and BRCA_EV^Lo^ tumors (mean ± SEM). ** *p* < 0.01, ***** *p* < 0.00001. (**I**) The anti-correlation of STAT3, IL6R, and IL6ST with EV score within non-metastatic breast cancer patients (ρ; Spearman correlation coefficient, line; linear correlation). (**J**) Spearman coefficient between genes of the STAT1 and STAT3 pathways and the EV score within non-metastatic breast cancer patients. (**K**) Volcano plot of overall survival of pan-cancers divided by the median expression of CD81^+^CD63^+^EVs gene signatures. (**L**) Overall survival of non-metastatic SKCM, LUSC and STES patients divided by median expression of CD81^+^CD63^+^EVs gene signatures.

Based on the mice data, we hypothesized that the inability to form tumors can be associated with immune response, and thus we sought to directly understand the impact of CD81^+^CD63^+^EVs and the link to the immune system within human patients with breast cancer. We analyzed the correlation between gene expression and survival of non-metastatic breast cancer patients available within The Cancer Genomic Atlas (TCGA). We first compared the survival of patients with higher and lower expressions of CD63 and CD81. Since there was no difference in survival of patients stratified by CD63 or CD81, we conclude that these single markers are necessary but not sufficient to identify a complex property such as EV secretion (Figure 5A and Figure S6A).

To identify signatures of CD81^+^CD63^+^EVs, we applied unsupervised hierarchal clustering to stratify non-metastatic breast cancer patients into two groups with 182 and 268 patients each (Figure S6B). A set of 13 genes related to EV secretion (CD81^+^CD63^+^EV signature) were identified as being differentially expressed between these two groups (Figure 5B). We, therefore, utilized the median expression of these genes to stratify patient tumors as CD81^+^CD63^+^EV high (BRCA_EV^Hi^) and low (BRCA_EV^Lo^). Consistent with our scRNA-seq data on 4T1 and 67NR cells, the expression of genes in the ALIX-Syndecan-Syntenin pathway was elevated in BRCA_EV^Hi^ patients in comparison with the BRCA_EV^Lo^ patients (Figure S6C). Simultaneously, the scRNA-seq data showed that 67NR cells are upregulated for the CD81^+^CD63^+^EV signature genes in comparison to 4T1 cells (Figure S6D). These results indicate that the genes identified in TCGA data can be applied as a signature for CD81^+^CD63^+^EVs. Evaluating the effect of CD81^+^CD63^+^EVs in the outcome of patients, the overall survival was significantly higher for BRCA_EV^Hi^ patients in comparison with the BRCA_EV^Lo^ patients (median survival not reached vs. 10.8 years, HR: 0.4, 95% CI: 0.18–0.92), consistent with our findings in mice that non-metastatic cells secreting CD81^+^CD63^+^EVs do not form tumors (Figure 5C and Figure 4E).

To identify if immune cell infiltration is associated with improved overall survival observed in patients with higher expression of CD81^+^CD63^+^EV, we used the previously published cytolytic score (based on the expression of GZMA and PRF1) as an in silico metric of immune cell cytolytic activity [43]. The cytolytic activity was significantly elevated in the BRCA_EV^Hi^ cohort compared with the BRCA_EV^Lo^ cohort (Figure 5D). To identify the immune cell type that was responsible for this signature, we used the normalized gene expression data to quantify the relative frequencies of the 22 different immune cell types using the CIBERSORTx algorithm [44]. CD8 T cells were not significantly different between the two clusters (Figure S6E). The difference in cytolytic activity was reflected with significant differences in macrophage subsets: a higher frequency of M0 and pro-inflammatory M1 macrophages, and a decreased frequency of anti-inflammatory M2 macrophages was observed in the tumors of BRCA_EV^Hi^ patients compared with the BRCA_EV^Lo^ patients (Figure 5E). Similar to the macrophages, the frequency of intratumoral memory CD4 T cells was also significantly different between BRCA_EV^Hi^ and BRCA_EV^Lo^ tumors. We utilized signatures of helper T cells within the previously described immunome signature set [45], to identify that Th1 cells were significantly increased, and Th17 cells were significantly decreased in the BRCA_EV^Hi^ patients compared with the BRCA_EV^Lo^ patients (Figure 5F). Collectively, these results showed that tumors in BRCA_EV^Hi^ patients harbor M0/M1 macrophages and Th1 cells.

We utilized GSEA to identify soluble mediators of the immune cell polarization within the tumor microenvironment of these patients. Not surprisingly, several pathways associated with chemokine/cytokine receptor interactions were enriched in BRCA_EV^Hi^ tumors (Figure 5G). Consistent with the high frequency of Th1 cells, interferon-gamma (IFN-γ) signaling was significantly elevated within the EV high tumors (Figure S6F). It is well known that the priming of macrophages in the presence of IFN-γ leads to the differentiation of pro-inflammatory M1 macrophages and downregulation of the IL6 signaling pathway. Although the expression of the IL6 receptor (IL6R) was not different, the expression of IL6 signal transducer (IL6ST) and the downstream signal transducer and activator of transcription 4 (STAT3) were significantly decreased in the BRCA_EV^Hi^ tumors compared with the BRCA_EV^Lo^ tumors (Figure 5H). We also utilized the median expression score of the 13 EV signature genes to confirm a significant inverse correlation between the EV signature and IL6R, IL6ST, and STAT3 within this entire cohort of patients (Figure 5I,J). Taken together, the secretion of CD81^+^CD63^+^EVs likely influences the infiltration of Th1 cells and a skewed ratio of M1/M2 macrophages through the crosstalk between IFN-γ and IL6/STAT3 pathways.

We investigated the utility of the EV secretion signature and its association with patient survival across pan-cancer datasets within the TCGA. Similar to breast cancer, EV secretion signatures were associated with improved overall survival in melanoma (SKCM) patients (14.3 vs. 9.4 years, HR: 0.62, 95% CI: 0.39–0.97) [Figure 5K]. By contrast, in both lung squamous cell carcinoma (LUSC) and stomach and esophageal carcinoma (STES), EV secretion signature was associated with worse overall survival for patients (Figure 5L).

## 4. Discussion

EV research is principally focused on the classification of EVs, studying the cargo of EVs, and elucidating the putative impact of EVs in cancer progression. For example, EVs derived from metastatic cancer cells have been proposed to induce a broad range of tumor-relevant functions: to remodel the extracellular matrix and the transformation of fibroblasts, promote angiogenesis, prepare the pre-metastatic niche, and alter the nature of the tumor microenvironment [46,47,48]. The relevance of these studies to actual cancer biology remains debatable because of the use of extraordinarily high concentrations of cell culture-derived EVs [4]. The approach of exogenous administration of supraphysiological levels of cell culture-derived EVs also masks the heterogeneity in EVs and the cargo they carry, and alters the functional impact on target cells. Single-vesicle profiling studies have elegantly demonstrated the heterogeneity in the surface markers and the cargo of EVs, but yet mapping the EV secretion onto individual cells remains challenging [49,50]. Recently, single-cell methods have reported the heterogeneity of EV secretion within cancer cells, but even in these studies, the ability to isolate and propagate cells with differences in EV secretion capabilities has not been illustrated [22,23,25]. We developed and validated a platform based on nanowell arrays for directly profiling EV secretion from single cells and used these to establish cells derived from a clinically relevant mouse breast cancer model that have significant differences in the rate of secretion of CD81^+^CD63^+^EVs.

We observed that surprisingly, the non-metastatic cell line, 67NR secretes more CD81^+^CD63^+^EVs per cell than its isogenic, metastatic counterpart, 4T1. To facilitate a deeper understanding of the impact of secretion of CD81^+^CD63^+^EVs, we tried to establish clonal sublines with the high and low secretion of CD81^+^CD63^+^EVs from both 67NR and 4T1 cells. We successfully established 67NR-S and 67NR-NS but were not able to establish a 4T1 cell line with high CD81^+^CD63^+^EV secretion suggesting that 4T1 cells do not likely produce high quantities of these EVs. To understand the implications of the data, we can combine the results of the mice studies we have undertaken with prior publications that have reported the exogenous bolus transfer of EVs from either 67NR or 4T1 cells [51]. Treatment of tumor-naïve mice with EVs from either 4T1 or 67NR cells showed significant biodistribution of the EVs in the lung and liver, consistent with the hypothesis that EVs seed the microenvironment at these tissues for subsequent metastasis [51]. Once 4T1 tumor cells were injected into animals, the immune cells infiltrate in the lung and liver (metastatic sites) showed an increase in the frequency of immunosuppressive monocytic myeloid-derived suppressor cells (M-MDSC) when comparing animals pretreated with 4T1 EVs to 67NR EVs. 4T1 cells that secrete low amounts of CD81^+^CD63^+^EVs foster a microenvironment comprised of immunosuppressive myeloid cells. In our animal studies, since the isogenic cell lines differed in the secretion capacity of CD81^+^CD63^+^EVs, we did not need to perform the supraphysiological transfer of EVs. In mice, 67NR-S cells were not able to form tumors, despite enhanced tumor-forming potential in vitro compared with 67NR-NS. Since there were no tumors available for profiling the role of the immune compartment, we used the data from the TCGA to investigate the role of the immune cells. Consistent with the observations in 4T1/67NR mouse models, breast tumors with signatures of low CD81^+^CD63^+^EVs had a significantly higher frequency of M2 macrophages (known to differentiate from M-MDSCs [52]) compared with tumors with signatures of high CD81^+^CD63^+^EVs. By identifying and implementing signatures of CD81^+^CD63^+^EVs within the TCGA, we were able to circumvent the differences in the immune microenvironment in mouse tumors such as 4T1 and human breast cancers. Thus, in human breast cancers, tumors with signatures of high CD81^+^CD63^+^EVs are more pro-inflammatory and associated with better clinical outcomes.

We also show that the CD81^+^CD63^+^EV secretion signatures were associated with improved overall survival in melanoma (SKCM) patients. These results are again consistent with the data from tumor experiments in mice using mouse (B16) melanoma cell lines [53]. The authors took advantage of the observation that Pigment epithelium-derived factor (PEDF) was differentially expressed in non-metastatic EVs (Exo^NM^) compared with metastatic EVs (Exo^M^). Consistent with our comparisons, both Exo^NM^ and Exo^M^ expressed CD63 and CD81. Exogenous transfer of Exo^NM^ inhibited lung colonization of mice that were administered B16F10 cells through the polarization of pro-inflammatory macrophages. These data from non-metastatic melanoma suggest that CD81^+^CD63^+^EVs can have a dominant effect in shaping the immune microenvironment leading to inhibition of metastasis. Both the breast cancer and melanoma studies have focused on the impact of CD81^+^CD63^+^EVs in inhibiting lung metastasis and it remains to be seen if this impact of CD81^+^CD63^+^EVs is restricted to the lung or if it also impacts metastasis to the other organs.

Although we have studied the role CD81^+^CD63^+^EVs in tumorigenicity and metastasis, we recognize that single-vesicle profiling studies have estimated that CD81^+^CD63^+^EVs comprise < 25 % of all EVs [49,50]. Similar studies will have to be undertaken to understand the impact of other subsets of EVs. We emphasize however that our high-throughput single-cell methodology can enable the isolation of isogenic cells with differences in EV secretion potential and enables studying the impact of EVs secreted at unmanipulated and physiological levels.

## 5. Conclusions

Our platform has direct utility in single-cell studies of profiling the link between EV secretion and function. First, the ability to isolate cells based on differences in either pan EVs or subsets of EVs as we demonstrated here can be utilized to perform scRNA-seq on the retrieved cells directly. This method will have great utility to map the molecular components of the EV secretion cascade directly. Second, based on the differentially expressed transcripts, it will be possible to infer the proteins that are likely enriched in the EVs secreted by these single cells. Third, the establishment of cell lines with differences in EV secretion among metastatic cells will help map the functional impact of EV secretion and their role in the biology of metastasis. We anticipate that our method can be broadly utilized to map the functional consequences of EVs secretion at the single-cell level.

## Data Availability

The Single Cell RNA sequencing data can be found at NCBI GEO data base with GEO association number GSE179115 (GEO Accession viewer (nih.gov).

## Supplementary Materials

The following are available online at https://www.mdpi.com/article/10.3390/cancers13174397/s1, Supplementary materials: Supplementary text, Video S1: Retrieving the single cells from nanowell using an automated micromanipulator. Figure S1: Characterization of EVs isolated by ultracentrifugation and bead-based immunoassays. Figure S2: Distribution of functionalized beads and pre-stained cells in individual nanowells. Figure S3: Overall workflow of the automated image-analysis for the identification of secretor and non-secretor cells. Figure S4: Transcriptome comparison of 67NR (non-metastatic) and 4T1 (metastatic) cells by single-cell and bulk RNA-seq. Figure S5: Single-cell comparison of 67NR-S and 67NR-NS cell lines for secretion of EVs subsets. Figure S6: The impact of secretion of CD81^+^CD63^+^EVs on non-metastatic human breast tumors.
