# Supplementary material for: Single-Cell Cloning of Breast Cancer Cells Secreting Specific Subsets of Extracellular Vesicles
Source: Cancers (Basel). 2021 Aug 31;13(17):4397. doi: 10.3390/cancers13174397 (PMC8430892; doi:10.3390/cancers13174397)
Supplement: Supplementary file 1 [file cancers-13-04397-s001.zip › Fathi_Cancers_SI_FINAL.pdf]

Supplementary Information

Single-Cell Cloning of Breast Cancer Cells Secreting Specific Subsets of Extracellular Vesicles

Mohsen Fathi [1], Robiya Joseph [2], Jay R. T. Adolacion [1,3], Melisa Martinez-Paniagua [1], Xingyue An [1], Konrad Gabrusiewicz [4], Sendurai A. Mani [2] and Navin Varadarajan [1,*]

[1] Chemical and Biomolecular Engineering Department, University of Houston, 4726 Calhoun Rd, Houston, TX 77204, USA; mfathi@uh.edu (M.F.); jtadolacion@up.edu.ph (J.R.T.A.); mamart51@Central.uh.edu (M.M.-P.); xingyuean@gmail.com (X.A.)

[2] Department of Translational Molecular Pathology, University of Texas M.D. Anderson Cancer Center, 2130 W Holcombe Blvd, Houston, TX 77030, USA; RJoseph7@mdanderson.org (R.J.); mani@mdanderson.org (S.A.M.)

[3] Department of Chemical Engineering, College of Engineering, University of the Philippines Diliman, Quezon City 1101, Philippines

[4] Department of Neurosurgery, University of Texas M.D. Anderson Cancer Center, 1400 Holcombe Blvd, Houston, TX 77030, USA; konrad.gabrusiewicz@gmail.com

* Correspondence: nvaradar@central.uh.edu; Tel.: +1-713-743-1691

This PDF file includes:

Supplementary text
Figures S1 to S6
Tables S1
Legends for Video S1
SI References

Material and Methods

FBS preparation

To prepare EV-free FBS, we used the ultracentrifugation procedure (Sorvall WX, ThermoFisher Scientific) by centrifuging the FBS at 100,000 x g for 18 hours (F37L-8 x 100 Fixed-Angle Rotor, Thermofisher Scientific).

EV isolation and measurement

We used ultracentrifugation (Sorvall WX, ThermoFisher Scientific) to isolate EVs. Starting with 250 ml of culture media, we centrifuged the conditioned media at 300 × g for 4 minutes, filtered with 0.22 µm filters, and centrifuged at 10,000 × g for 30 minutes followed by ultracentrifugation at 100,000 × g for 70 minutes to pellet the EVs (F37L-8 x 100 Fixed-Angle Rotor, Thermofisher Scientific). We washed the EVs pellet with PBS twice and centrifuged for another 100,000 × g for 70 minutes to purify the EVs (TH-660 swinging bucket rotor, Thermofisher Scientific). We resuspended the EVs in PBS and measured the EV size distribution using the nanoparticle tracking analyzer (Nanosight LM10-HS, Nanosight Ltd, Malvern Instrument). We stored the isolated EVs at 4°C for one week or at -80°C for long term use.

EV quantification using transwell assay

We utilized a Transwell insert with 3 µm pore membrane and loaded functionalized beads at the lower compartment, and cells on the upper compartment of the insert. For the GW4869 treatment assay, we used EV-free complete media containing either 10 µM GW4869 or 0.2% DMSO. After 48 hours of incubation at 37°C, we collected the beads and labeled them with 4 µg/ml PE anti-CD63 antibody (BioLegend, catalog number 143903 - clone NVG-2) for 45 minutes at 37°C. We subsequently washed the beads three times in PBS with 1% BSA and performed imaging using a Zeiss Axio Observer Z1

microscope equipped with 20x/0.8 NA objectives. Using ImageJ, we segmented and measured the fluorescent intensity of CD63 on the beads.

Transmission Electron Microscopy (TEM)

Via EV quantification using a transwell assay, and after 48 hours incubation at 37°C, we collected the beads and fixed with 2% glutaraldehyde (Ladd research, catalog number 20215). We placed the samples on 100-mesh carbon-coated, formvar-coated copper grids treated with poly-L-lysine for approximately 1 hour. We then negatively stained the samples with Millipore-filtered aqueous 1% uranyl acetate for 1 minute. The stain was blotted dry from the grids with filter paper, and samples were allowed to dry. We examined the samples in a JEM 1010 transmission electron microscope (JEOL, USA, Inc., Peabody, MA) at an accelerating voltage of 80 kV. We obtained the digital images were using the AMT Imaging System (Advanced Microscopy Techniques Corp., Danvers, MA).

PDMS nanowell array fabrication and preparation

Applying standard soft lithography techniques, we fabricated the PDMS nanowell array as previously described [1]. Before loading cells on the nanowell, we re-oxidized the array with air plasma and incubated with 1.5 ml PLL-g-PEG (SuSoS, Switzerland) solution dissolved in 10 mM HEPES buffer for 20 minutes at 37°C. After incubation, we rinsed the array with complete media before loading the cells.

Wound healing assay

We cultured 67NR-S and 67NR-NS cells in a 12-well plate to 90% confluency with 10% FBS complete media. We subsequently replaced the media with 0.5% EV-free complete media for 12 hours. After starvation, we scratched the cells with 10 μl pipette tips and washed twice with PBS to remove the detached cells. We cultured the cells with 0.5% EV-free complete media during the assay to slow down cell proliferation. We obtained the images from six different areas per well with Zeiss Axio Observer Z1 microscope equipped

with 20x/0.5 NA objectives at several time points. We analyzed the images with TScratch tool [2].

Soft agar colony formation assay

Performing an anchorage-independent growth assay using SeaPlaque agarose (Lonza, catalog number 50101), we assessed the transformation capacity of the 67NR-S and 67NR-NS cells *in vitro*. We used three different conditions in triplicates to determine the ability of these cells to form soft agar colonies: no treatment, 0.2% DMSO, and 10 µM GW4869 (Cayman Chemical, catalog number 13127). We suspended 2.5×10^3 cells in 0.7% top agar in EV-free complete media containing the appropriate treatment conditions and placed on top of solidified 0.8% bottom agar in 6-well plates (Fisher, catalog number 353046). Upon setting of the top agar with cells, we added 500 µl of fresh EV-free complete media containing the appropriate treatment conditions to the wells and incubated the plates for 14 days at 37°C. We fed the cells with EV-free complete media with the appropriate treatments, twice per week. We counted the colonies from ten different areas per well and acquired the representative 20x images microscopically using Zeiss Axio Observer A1 microscope.

Bulk RNA sequencing dataset analysis

We downloaded the raw counts of RNA-seq dataset published by Kim et al. from GEO (GSE104765) [3]. We filtered the table for three replicates of 4T1 and 67NR cells. To obtain the differentially expressed genes, we used the DESeq2 (v1.22.2) package [4] in R.

Single-cell RNA-sequencing

Following the Illumina Bio-Rad SureCell WTA 3' library prep reference guide, we prepared the scRNA-seq library. Briefly, we mixed an equal number of the mouse cell lines in cold PBS with 0.1% BSA in a concentration of 2,500 cells/µl, then filtered to achieve

single-cell suspension. Using a ddSEQ Single-Cell Isolator, we co-encapsulated with oil the single cells and barcodes into droplets. After reverse transcribing and breaking the emulsion, we purified the first-strand products using purification beads, followed by cDNA synthesis and tagmentation. We PCR-amplified the cDNA and cleaned it up to remove short library fragments. Later, we sequenced the cDNA library in a NextSeq 500 sequencing system. Using Illumina BaseSpace Sequence Hub, we analyzed the sequencing data and created a count matrix containing the number of transcriptomes for every single cell. We imported these matrices into R and combined them into a single matrix, which was then cleaned, normalized, and analyzed using the Seurat (v3.1.4) package [5]. We ranked the differentially expressed genes of 4T1 and 67NR cell lines and transformed into human orthologous using the BiomaRt (v2.38.0) package [6,7], and imported to GSEA software [8,9] provided by UC San Diego and Broad Institute for gene set enrichment analysis.

In vivo **studies**

We injected 1×10^4 67NR-S and 67NR-NS cells subcutaneously into the fourth left mammary fat pad of five BALB/c mice (Jackson laboratory, strain 0000651 BALB/cJ) for each clone. We monitored the size of the tumor with caliper measurements weekly and calculated using formula $(L \times W^2) \times 0.5$, where L and W are the length and the width of the tumor, respectively. We sacrificed the mice and harvested the tumors before the onset of necrosis.

Tumor Cancer Genome Atlas (TCGA) data processing

We downloaded all the TCGA data, including raw counts, RSEM gene normalized expression, and clinical data from the Broad Institute FireBrowse Data Portal (www.firebrowse.org). To collect the non-metastatic patients without lymph node metastasis, we used the TNM staging information and selected the patients with N0 and

M0 for analysis. Subsequently, we removed the genes with average RSEM expression < 5 from analysis.

EV signature identification in Tumor Cancer Genome Atlas (TCGA) dataset

To identify a signature gene associated with $CD81^{+}CD63^{+}$EVs, we perform hierarchal clustering on the non-metastatic breast cancer dataset. We used *hclust* function in R to identify two clusters using *ward.D2* as the linkage method with *manhattan* as the distance measure (Figure S6B). Using the DESeq2 (v1.22.2) package, we identified CD63 and CD81 upregulated in cluster 1. Using the set of genes associated with EV secretion (Table S1), we identified 13 genes with more than 1.2-fold change in cluster 1 as $CD81^{+}CD63^{+}$ EVs signature genes for further analysis (Figure 5B).

Data mining in Tumor Cancer Genome Atlas (TCGA) dataset

For gene set enrichment analysis, we used the pre-ranked gene list of genes with a significant fold change ($p < 0.05$) in GSEA software provided by UC San Diego and Broad Institute. For survival analysis, we used the Kaplan-Meier method to compare the overall survival of patients divided by the median expression of $CD81^{+}CD63^{+}$EVs signature genes. We tested the statistical significance of survival curves using the log-rank test. We calculated the cytolytic activity (Cyt) as the geometric mean of PRF1 and GZMA as previously described [10]. For immune cell infiltration estimation, we performed CIBERSORTx [11] analysis on the RSEM gene expression of breast cancer patients to estimate the relative fraction of 22 immune cell types using 1000 permutations. To calculate the ssGSEA score of immune cells, we used GSVA (v1.30.0) package [12] using gene signatures collected from a previously described signature [13]. Using *cor* function in R, we calculated the Spearman's rank correlation between the median expression of $CD81^{+}CD63^{+}$EVs signature genes (EVs score) and a single gene of interest.

Statistical analysis

All data presented represent mean ± SEM, unless mentioned. We performed two-tailed t-test between two groups, unless indicated otherwise. For correlation analysis, we performed nonparametric Spearman rank correlation. We performed two-way ANOVA test to evaluate the difference between samples in wound healing assay. Analyses were carried out in GraphPad Prism (v6.0). We considered p value < 0.05 as a significant threshold for comparisons, unless indicated otherwise.

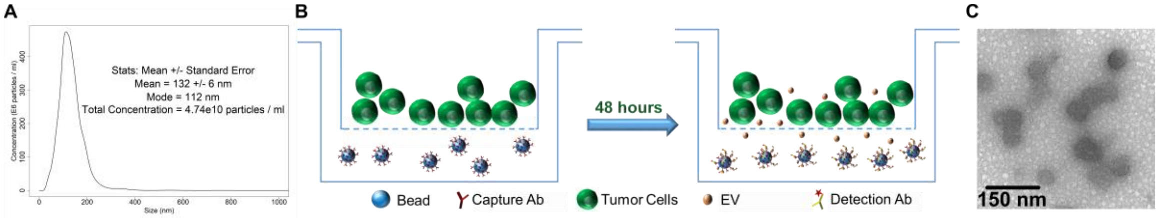

Figure S1. Characterization of EVs isolated by ultracentrifugation and bead-based immunoassays.

A. Nanosight analysis of EVs isolated from cancer cells.

B. Schematic of transwell assay for capturing EVs using immunoassay.

C. TEM images of EVs isolated using the transwell assay.

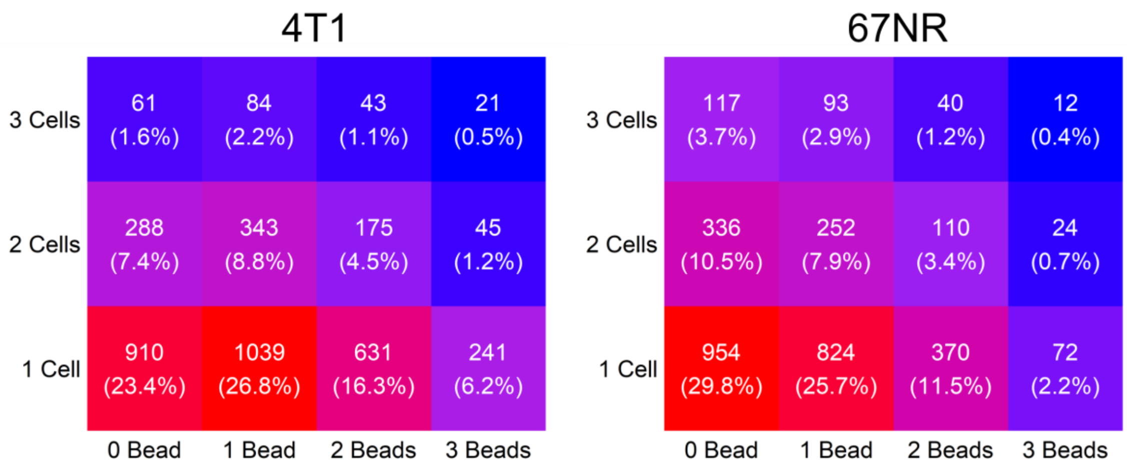

Figure S2. Distribution of functionalized beads and pre-stained cells in individual nanowells. Two representative density matrixes, 4T1 and 67NR, indicate the number of nanowells that contain 0-3 beads and 1-3 cells.

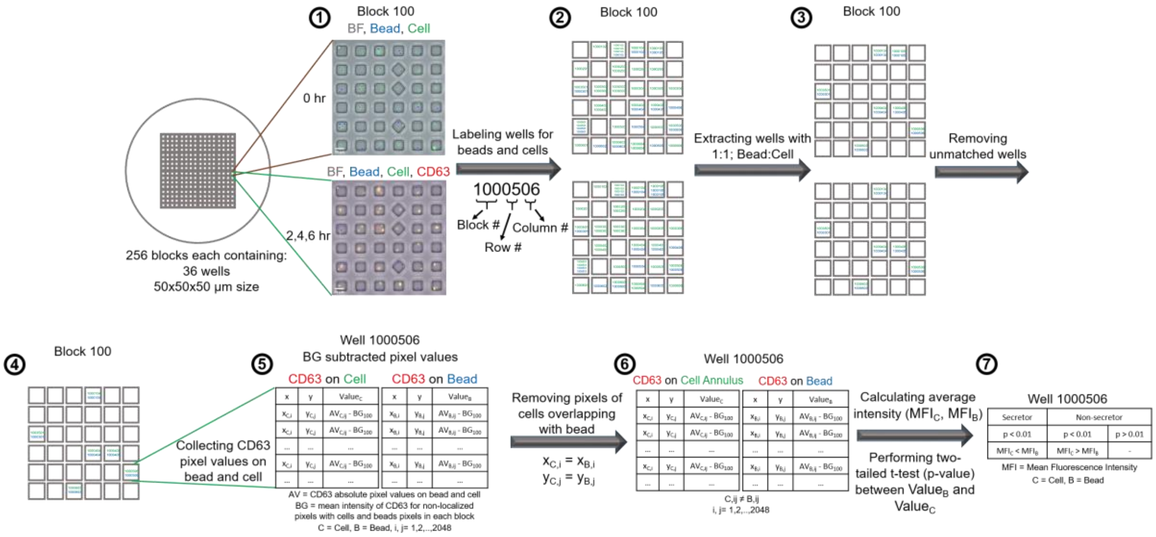

Figure S3. Overall workflow of the automated image-analysis for the identification of secretor and non-secretor cells.

1. TIFF images of 256 field of views (blocks) containing 36 wells were extracted and merged for the initial time point (0 hour) and the detection time points (2, 4, 6 hours). Block 100 is shown as an example for workflow.

2. The numbers of beads and cells in each well were identified for each block at all the time points using *analyze particles* function in ImageJ.

3. Wells with a single bead and a single cell were identified.

4. The wells that did not have a bead:cell = 1:1 ratio for the entire duration of experiment were excluded from the further analysis.

5. The CD63 pixel values on the surface of cell and bead were determined. These values were corrected using background subtractions. To calculate background intensity, the average of CD63 pixel values for each block was calculated (excluding the pixels corresponding to cells and beads). Well 1000506 is shown as an example for workflow.

6. We identified the pixels corresponding to the cell and the bead separately. The pixels that were common between bead and cell were removed from pixel value sets of the cell. This created an annulus shape for the pixels on the surface of cell. A two-tailed t-test was applied on two set of pixel values, those corresponding to the bead and the other corresponding to cell annulus. If the pixel values on the beads were significantly ($p < 0.01$) higher than cell annulus intensity, the cell was classified as a secretor.

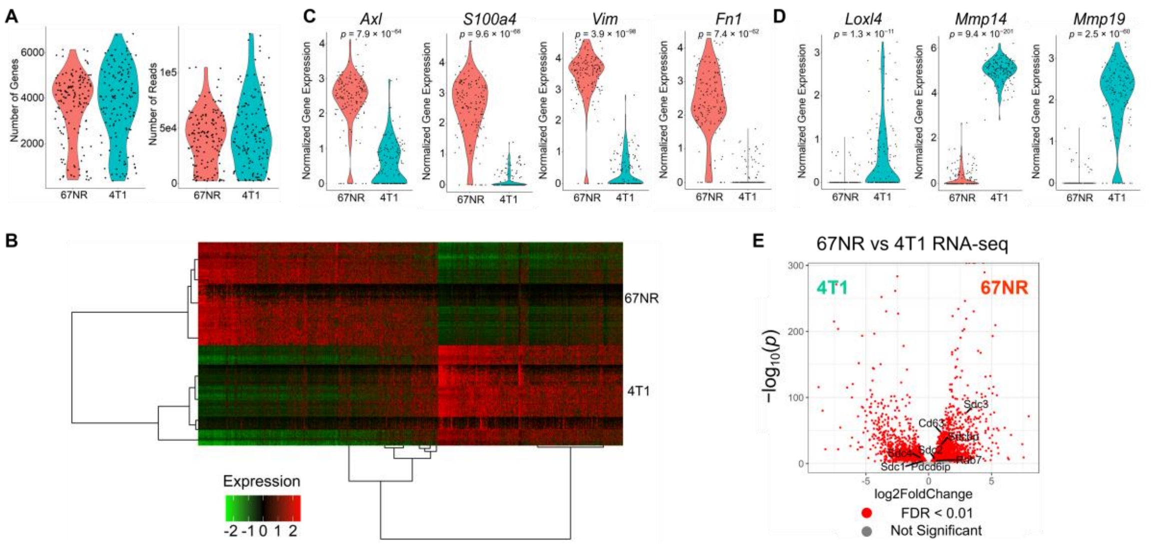

Figure S4. Transcriptome comparison of 67NR (non-metastatic) and 4T1 (metastatic) cells by single-cell and bulk RNA-seq.

A. The number of genes and reads detected in scRNA-seq for 67NR and 4T1 cells.

B. Heat map comparing the expression of the differentially expressed genes in 67NR and 4T1 cells.

C. Violin plot comparing the expression of mesenchymal cell transcripts.

D. Violin plot comparing the expression of epithelial cell transcripts.

E. Expression of genes associate with Alix-Syndecan-Syntenin pathway in 67NR cells in comparison with 4T1 cells based on the bulk RNA sequencing.

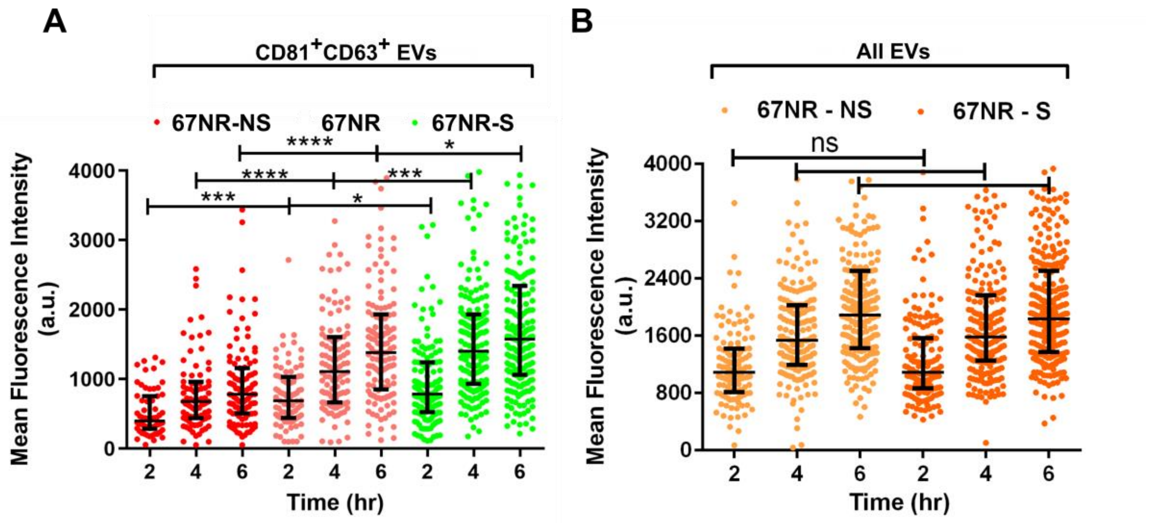

Figure S5. Single-cell comparison of 67NR-S and 67NR-NS cell lines for secretion of EVs subsets.

A. Higher and lower secretion rate of $CD81^+CD63^+$EVs for 67NR-S and 67NR-NS cells in comparison to parental 67NR cells, respectively. Each dot represents a single cell with the median and quartiles of CD63 (EVs) intensity shown over all cells. * $p < 0.01$, *** $p < 0.0001$, **** $p < 0.00001$.

B. Secretion rate of pan EVs identified by combination of CD63, CD81, and CD9 surface markers. Each dot represents a single cell with the mean fluorescence intensity of PE anti-CD63, PE anti-CD81, and PE anti-CD9 antibodies shown on the y-axis. ns: nonsignificant.

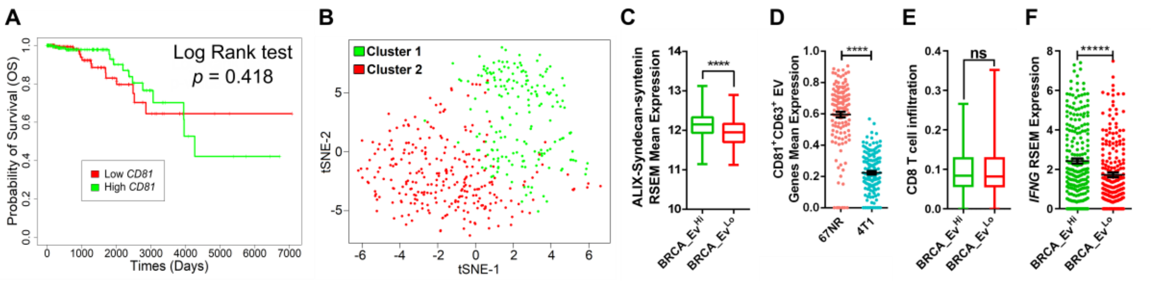

Figure S6. The impact of secretion of CD81⁺CD63⁺EVs on non-metastatic human breast tumors.

A. Overall survival of non-metastatic breast cancer patients (N0 and M0 in TNM staging system) divided by CD81 median expression.

B. T-SNE plot of the clusters identified by unsupervised hierarchal clustering of non-metastatic breast cancer patients.

C. The average expression of genes in ALIX-Syndecan-Syntenin pathway (*CD63*, *SDC1*, *SDC2*, *SDC3*, *SDC4*, *SDCBP*, *RAB7A*, *PDCD6IP*) within BRCA_EV^{Hi} and BRCA_EV^{Lo} patients (mean ± SEM). **** $p < 0.0001$.

D. The average expression of CD81^{+}CD63^{+}EV signature genes within 67NR and 4T1 cells profiled by scRNA-seq analysis (mean ± SEM). **** $p < 0.0001$.

E. Infiltration of CD8 T cells within BRCA_EV^{Hi} and BRCA_EV^{Lo} patients (mean ± SEM). The two-tailed t-test was used for comparison. ns: nonsignificant.

F. High expression of *IFNG* in BRCA_EV^{Hi} patients in comparison to BRCA_EV^{Lo} patients (mean ± SEM). ***** $p < 0.00001$.

Table S1. Gene signature associated with EV secretion.

	Mouse symbol	Human Symbol	Ref.		Mouse symbol	Human Symbol	Ref.
1	*Hgs*	*HGS*	14-17	22	*Pkm*	*PKM*	18
2	*Stam*	*STAM*	14	23	*Snap23*	*SNAP23*	18
3	*Tsg101*	*TSG101*	14	24	*Rala*	*RALA*	19
4	*Chmp4c*	*CHMP4C*	14	25	*Ralb*	*RALB*	19
5	*Pdcd6ip*	*PDCD6IP*	14,20	26	*Rab2b*	*RAB2B*	21
6	*Vta1*	*VTA1*	14	27	*Rab5a*	*RAB5A*	21
7	*Vps4a*	*VPS4A*	14	28	*Rab9*	*RAB9A*	21
8	*Sdcbp*	*SDCBP*	20	29	*Rab7*	*RAB7A*	20,22
9	*Sdc1*	*SDC1*	20	30	*Rab11a*	*RAB11A*	23,24
10	*Sdc2*	*SDC2*	20	31	*Rab27a*	*RAB27A*	17,21,25-27
11	*Sdc3*	*SDC3*	20	32	*Rab27b*	*RAB27B*	21,22
12	*Sdc4*	*SDC4*	20	33	*Rab35*	*RAB35*	28,29
13	*Cd9*	*CD9*	30	34	*Cit*	*CIT*	31
14	*Cd82*	*CD82*	30	35	*Cttn*	*CTTN*	32
15	*Cd63*	*CD63*	33	36	*Smpd3*	*SMPD3*	34-36
16	*Lmp1*	*LMP1*	37	37	*Dgka*	*DGKA*	38
17	*Tspan8*	*TSPAN8*	39	38	*Pld2*	*PLD2*	40,41
18	*Syt7*	*SYT7*	17	39	*Arf6*	*ARF6*	41
19	*Vamp7*	*VAMP7*	42	40	*Bst2*	*BST2*	43,44
20	*Ykt6*	*YKT6*	16,45	41	*Atg12*	*ATG12*	46
21	*Stx1a*	*STX1A*	24	42	*Atg3*	*ATG3*	46

Video S1 (separate file). Retrieving the single cells from nanowell using an automated micromanipulator.

SI References

1. An, X., Sendra, V. G., Liadi, I., Ramesh, B., Romain, G., et al (2017). Single-cell profiling of dynamic cytokine secretion and the phenotype of immune cells. *PLoS One*, *12*(8), e0181904.
2. Gebäck, T., Schulz, M. M. P., Koumoutsakos, P., & Detmar, M. (2009). TScratch: A novel and simple software tool for automated analysis of monolayer wound healing assays: Short Technical Reports. *Biotechniques*, *46*(4), 265-274.
3. Kim, I. S., Gao, Y., Welte, T., Wang, H., Liu, J., et al (2019). Immuno-subtyping of breast cancer reveals distinct myeloid cell profiles and immunotherapy resistance mechanisms. *Nature cell biology*, *21*(9), 1113-1126.
4. Love, M. I., Huber, W., & Anders, S. (2014). Moderated estimation of fold change and dispersion for RNA-seq data with DESeq2. *Genome biology*, *15*(12), 1-21.
5. Stuart, T., Butler, A., Hoffman, P., Hafemeister, C., Papalexi, E., et al (2019). Comprehensive integration of single-cell data. *Cell*, *177*(7), 1888-1902.
6. Durinck, S., Moreau, Y., Kasprzyk, A., Davis, S., De Moor, B., et al (2005). BioMart and Bioconductor: a powerful link between biological databases and microarray data analysis. *Bioinformatics*, *21*(16), 3439-3440.
7. Durinck, S., Spellman, P. T., Birney, E., & Huber, W. (2009). Mapping identifiers for the integration of genomic datasets with the R/Bioconductor package biomaRt. *Nature protocols*, *4*(8), 1184-1191.
8. Subramanian, A., Tamayo, P., Mootha, V. K., Mukherjee, S., Ebert, B. L., et al (2005). Gene set enrichment analysis: a knowledge-based approach for interpreting genome-wide expression profiles. *Proceedings of the National Academy of Sciences*, *102*(43), 15545-15550.
9. Mootha, V. K., Lindgren, C. M., Eriksson, K. F., Subramanian, A., Sihag, S., et al (2003). PGC-1α-responsive genes involved in oxidative phosphorylation are coordinately downregulated in human diabetes. *Nature genetics*, *34*(3), 267-273.
10. Rooney, M. S., Shukla, S. A., Wu, C. J., Getz, G., & Hacohen, N. (2015). Molecular and genetic properties of tumors associated with local immune cytolytic activity. *Cell*, *160*(1-2), 48-61.
11. Newman, A. M., Liu, C. L., Green, M. R., Gentles, A. J., Feng, W., et al (2015). Robust enumeration of cell subsets from tissue expression profiles. *Nature methods*, *12*(5), 453-457.
12. Hänzelmann, S., Castelo, R., & Guinney, J. (2013). GSVA: gene set variation analysis for microarray and RNA-seq data. *BMC bioinformatics*, *14*(1), 1-15.
13. Bindea, G., Mlecnik, B., Tosolini, M., Kirilovsky, A., Waldner, M., et al (2013). Spatiotemporal dynamics of intratumoral immune cells reveal the immune landscape in human cancer. *Immunity*, *39*(4), 782-795.
14. Colombo, M., Moita, C., Van Niel, G., Kowal, J., Vigneron, J., et al (2013). Analysis of ESCRT functions in exosome biogenesis, composition and secretion highlights the heterogeneity of extracellular vesicles. *Journal of cell science*, *126*(24), 5553-5565.
15. Tamai, K., Tanaka, N., Nakano, T., Kakazu, E., Kondo, Y., et al (2010). Exosome secretion of dendritic cells is regulated by Hrs, an ESCRT-0 protein. *Biochemical and biophysical research communications*, *399*(3), 384-390.
16. Gross, J. C., Chaudhary, V., Bartscherer, K., & Boutros, M. (2012). Active Wnt proteins are secreted on exosomes. *Nature cell biology*, *14*(10), 1036-1045.

17. Hoshino, D., Kirkbride, K. C., Costello, K., Clark, E. S., Sinha, S., et al (2013). Exosome secretion is enhanced by invadopodia and drives invasive behavior. *Cell reports*, *5*(5), 1159-1168.
18. Wei, Y., Wang, D., Jin, F., Bian, Z., Li, L., et al (2017). Pyruvate kinase type M2 promotes tumour cell exosome release via phosphorylating synaptosome-associated protein 23. *Nature communications*, *8*(1), 1-12.
19. Hyenne, V., Apaydin, A., Rodriguez, D., Spiegelhalter, C., Hoff-Yoessle, S., et al (2015). RAL-1 controls multivesicular body biogenesis and exosome secretion. *Journal of Cell Biology*, *211*(1), 27-37.
20. Baietti, M. F., Zhang, Z., Mortier, E., Melchior, A., Degeest, G., et al (2012). Syndecan–syntenin–ALIX regulates the biogenesis of exosomes. *Nature cell biology*, *14*(7), 677-685.
21. Ostrowski, M., Carmo, N. B., Krumeich, S., Fanget, I., Raposo, G., et al (2010). Rab27a and Rab27b control different steps of the exosome secretion pathway. *Nature cell biology*, *12*(1), 19-30.
22. Jaé, N., McEwan, D. G., Manavski, Y., Boon, R. A., & Dimmeler, S. (2015). Rab7a and Rab27b control secretion of endothelial microRNA through extracellular vesicles. *FEBS letters*, *589*(20), 3182-3188.
23. Savina, A., Vidal, M., & Colombo, M. I. (2002). The exosome pathway in K562 cells is regulated by Rab11. *Journal of cell science*, *115*(12), 2505-2515.
24. Koles, K., Nunnari, J., Korkut, C., Barria, R., Brewer, C., et al (2012). Mechanism of evenness interrupted (Evi)-exosome release at synaptic boutons. *Journal of Biological Chemistry*, *287*(20), 16820-16834.
25. Peinado, H., Alečković, M., Lavotshkin, S., Matei, I., Costa-Silva, B., et al (2012). Melanoma exosomes educate bone marrow progenitor cells toward a pro-metastatic phenotype through MET. *Nature medicine*, *18*(6), 883-891.
26. Bobrie, A., Krumeich, S., Reyal, F., Recchi, C., Moita, L. F., et al (2012). Rab27a supports exosome-dependent and-independent mechanisms that modify the tumor microenvironment and can promote tumor progression. *Cancer research*, *72*(19), 4920-4930.
27. Webber, J. P., Spary, L. K., Sanders, A. J., Chowdhury, R., Jiang, W. G., et al (2015). Differentiation of tumour-promoting stromal myofibroblasts by cancer exosomes. *Oncogene*, *34*(3), 290-302.
28. Hsu, C., Morohashi, Y., Yoshimura, S. I., Manrique-Hoyos, N., Jung, S., et al (2010). Regulation of exosome secretion by Rab35 and its GTPase-activating proteins TBC1D10A–C. *Journal of Cell Biology*, *189*(2), 223-232.
29. Frühbeis, C., Fröhlich, D., Kuo, W. P., Amphornrat, J., Thilemann, S., et al (2013). Neurotransmitter-triggered transfer of exosomes mediates oligodendrocyte–neuron communication. *PLoS biology*, *11*(7), e1001604.
30. Chairoungdua, A., Smith, D. L., Pochard, P., Hull, M., & Caplan, M. J. (2010). Exosome release of β-catenin: a novel mechanism that antagonizes Wnt signaling. *Journal of Cell Biology*, *190*(6), 1079-1091.
31. Loomis, R. J., Holmes, D. A., Elms, A., Solski, P. A., Der, C. J., et al (2006). Citron kinase, a RhoA effector, enhances HIV-1 virion production by modulating exocytosis. *Traffic*, *7*(12), 1643-1653.
32. Sinha, S., Hoshino, D., Hong, N. H., Kirkbride, K. C., Grega-Larson, N. E., et al (2016). Cortactin promotes exosome secretion by controlling branched actin dynamics. *Journal of Cell Biology*, *214*(2), 197-213.

33. Hurwitz, S. N., Conlon, M. M., Rider, M. A., Brownstein, N. C., & Meckes Jr, D. G. (2016). Nanoparticle analysis sheds budding insights into genetic drivers of extracellular vesicle biogenesis. *Journal of extracellular vesicles*, *5*(1), 31295.
34. Trajkovic, K., Hsu, C., Chiantia, S., Rajendran, L., Wenzel, D., et al (2008). Ceramide triggers budding of exosome vesicles into multivesicular endosomes. *Science*, *319*(5867), 1244-1247.
35. Kosaka, N., Iguchi, H., Yoshioka, Y., Takeshita, F., Matsuki, Y., et al (2010). Secretory Mechanisms and Intercellular Transfer of MicroRNAs in Living Cells*♦. *Journal of Biological Chemistry*, *285*(23), 17442-17452.
36. Mittelbrunn, M., Gutiérrez-Vázquez, C., Villarroya-Beltri, C., González, S., Sánchez-Cabo, F., et al (2011). Unidirectional transfer of microRNA-loaded exosomes from T cells to antigen-presenting cells. *Nature communications*, *2*(1), 1-10.
37. Hurwitz, S. N., Nkosi, D., Conlon, M. M., York, S. B., Liu, X., et al (2017). CD63 regulates Epstein-Barr virus LMP1 exosomal packaging, enhancement of vesicle production, and noncanonical NF-κB signaling. *Journal of virology*, *91*(5), e02251-16.
38. Alonso, R., Mazzeo, C., Rodríguez, M. C., Marsh, M., Fraile-Ramos, A., et al (2011). Diacylglycerol kinase α regulates the formation and polarisation of mature multivesicular bodies involved in the secretion of Fas ligand-containing exosomes in T lymphocytes. *Cell Death & Differentiation*, *18*(7), 1161-1173.
39. Nazarenko, I., Rana, S., Baumann, A., McAlear, J., Hellwig, A., et al (2010). Cell surface tetraspanin Tspan8 contributes to molecular pathways of exosome-induced endothelial cell activation. *Cancer research*, *70*(4), 1668-1678.
40. Laulagnier, K., Grand, D., Dujardin, A., Hamdi, S., Vincent-Schneider, H., et al (2004). PLD2 is enriched on exosomes and its activity is correlated to the release of exosomes. *FEBS letters*, *572*(1-3), 11-14.
41. Ghossoub, R., Lembo, F., Rubio, A., Gaillard, C. B., Bouchet, J., et al (2014). Syntenin-ALIX exosome biogenesis and budding into multivesicular bodies are controlled by ARF6 and PLD2. *Nature communications*, *5*(1), 1-12.
42. Fader, C. M., Sánchez, D. G., Mestre, M. B., & Colombo, M. I. (2009). TI-VAMP/VAMP7 and VAMP3/cellubrevin: two v-SNARE proteins involved in specific steps of the autophagy/multivesicular body pathways. *Biochimica et Biophysica Acta (BBA)-Molecular Cell Research*, *1793*(12), 1901-1916.
43. Fu, Y., Zhang, L., Zhang, F., Tang, T., Zhou, Q., et al (2017). Exosome-mediated miR-146a transfer suppresses type I interferon response and facilitates EV71 infection. *PLoS pathogens*, *13*(9), e1006611.
44. Edgar, J. R., Manna, P. T., Nishimura, S., Banting, G., & Robinson, M. S. (2016). Tetherin is an exosomal tether. *Elife*, *5*, e17180.
45. Ruiz-Martinez, M., Navarro, A., Marrades, R. M., Viñolas, N., Santasusagna, S., et al (2016). YKT6 expression, exosome release, and survival in non-small cell lung cancer. *Oncotarget*, *7*(32), 51515.
46. Murrow, L., Malhotra, R., & Debnath, J. (2015). ATG12–ATG3 interacts with Alix to promote basal autophagic flux and late endosome function. *Nature cell biology*, *17*(3), 300-310.
